# Should fiscal policies be centralized in a monetary union? A dynamic game approach

**DOI:** 10.1007/s10100-023-00846-4

**Published:** 2023-03-14

**Authors:** Dmitri Blueschke, Viktoria Blueschke-Nikolaeva, Reinhard Neck

**Affiliations:** Department of Economics, University of Klagenfurt, Universitaetsstr. 65-67, 9020 Klagenfurt, Austria

**Keywords:** Dynamic game, Feedback Nash equilibrium, Pareto solution, Monetary union, Public debt, Coalitions

## Abstract

In this paper we analyze dynamic interactions in a monetary union with three fiscal players (the governments of the countries concerned) and a common central bank in the presence of exogenous shocks. The model is calibrated for the euro area and includes a fiscally more solid core block denoted as country 1 as well as a fiscally less solid periphery block represented by countries 2 and 3. Introducing two periphery countries allows us to capture different attitudes of the periphery countries towards the goal of sustainable fiscal performance. Moreover, different coalition scenarios are modelled in this study including a fiscal union, a coalition of periphery countries and a coalition of fiscal-stability oriented countries. The exogenous shocks are calibrated in such a way as to describe the last major crises in the euro area, namely the financial crisis, the European sovereign debt crisis, the Covid-19 crisis, and the Ukraine war (energy price) crisis. Using the OPTGAME algorithm we calculate a cooperative Pareto and non-cooperative feedback Nash equilibrium solutions for the modelled scenarios. The fully cooperative solution yields the best results. The different non-cooperative scenarios allow insights into the underlying trade-off between economic growth, price stability and fiscal stability.

## Introduction

In the last 14 years the euro area has been hit by four successive crises: the financial crisis 2008–2010, the European sovereign debt crisis, the Covid-19 crisis, and the Ukraine war (energy price) crisis. The financial crisis and the Covid-19 crisis are worldwide crises and most countries reacted to them using a countercyclical fiscal and monetary policy mix. The situation in the euro area is more complicated due to the additional sovereign debt crisis and the fact that the European Central Bank (ECB) shapes its monetary policy for all participating countries despite certain asymmetries between them. In order to take (some of) the existing differences between euro area economies into account, we propose to employ the game-theoretic framework to discuss the optimal design of fiscal and monetary policy in the euro area in the presence of a crisis.

Game theory is a natural way to analyze the dynamics inside a monetary union as it allows us to consider the strategic interactions of heterogeneous players. We do not restrict ourselves to the interaction between monetary and fiscal players—a topic already intensively discussed in the literature (see e.g. Nordhaus et al. 1994 for a survey)—but model a monetary union with three fiscal players (representing blocks of countries) and a common central bank to capture some specific asymmetries between the countries. Splitting the euro area countries into a core and a periphery block is widespread and has been analyzed in previous research, for example by Blueschke and Neck (2011), Neck and Blueschke (2014), Neck and Blueschke (2020) and Anastasiou et al. (2019). In this study we follow Blueschke and Neck (2018) and consider three fiscal players, which are calibrated in such a way so as to represent blocks of countries in the euro area, namely the core block (also called country 1) with relatively solid public finances, the thrifty periphery block (called country 2) with higher initial public debt but according relatively high importance to fiscal objectives and the thriftless periphery block with higher initial public debt and according little importance to fiscal targets (country 3).

Of course, the euro area consists of more countries but the analysis of interactions between all of them would be rather cumbersome without adding much to the question we investigate here. On the other hand, a severe problem arises from our assumption that all blocks are homogeneous and countries stay together in the respective coalition over the entire period of investigation. In contrast, in reality groups of countries (or single countries) and their governments may change, by countries leaving a coalition and switching to another coalition or staying alone over some subperiod. This may especially (but not only) occur when governments change due to an election. Another possibility is for a country to stay formally within the coalition but deviate from the course of policy agreed upon at the start of the game, for instance when a country wants to free ride to improve its position at the expense of the other members of the coalition. This would entail a complete re-calibration of the model, with at least as many countries as there are members of the monetary union–a number that even changes for the euro area during the period under consideration.

An additional difficulty would be due to the requirement to allow for changes in coalition structures during the game, which would necessitate a consideration of the incentives to enter or exit a coalition at any intermediate point of time, that is, to endogenize coalition formation. Coalition structures in a monetary union with endogenous coalition formation were studied earlier by Michalak et al. (2008) and in the references given there; see also Plasmans et al. (2006). This work uses a continuous-time model of a three-country monetary union and assumes a particular strategic interaction pattern of the countries involved. It may be a starting point to extend the present analysis to a more realistic model of the euro area. Interpreting our study in terms of the eurozone therefore should be done with care, considering these additional possibilities.

Altogether we consider a monetary union with four players. This allows us to analyze different coalition scenarios in a monetary union model for the euro area affected by the financial crisis 2008–2010, the European sovereign debt crisis, the Covid-19 crisis, and the Ukraine war crisis. The first two are modelled as pure demand-side shocks while the latter two also contain some supply-side elements. These shocks impact on a dynamic macroeconomic model of a monetary union. The governments of the three member countries of the union design their fiscal policies to optimize their (nationally determined) objective functions. In addition, the joint central bank of the union optimizes a (union-wide) objective function over the same model. All four players take account of the other players’ strategies, either in a noncooperative way according to the feedback Nash equilibrium solution concept or in a cooperative way with each other or with subsets of the other players by forming coalitions. The goal of the analysis is to obtain insights into the possible advantages of a more centralized design for fiscal policies in a monetary union. Throughout it is assumed that the objectives of the policy makers coincide with those of the citizens of the countries; hence no public-choice (political economy) considerations are involved.

The structure of the paper is as follows: Sect. 2 introduces the framework of dynamic game theory and the solution algorithm OPTGAME. Section 3 describes the model of the monetary union used in the analysis as well as the objective functions of the policy makers and specifies the numerical values of the parameters. In Sect. 4, the exogenous shocks are shown. The results of the game experiments with five scenarios are presented and interpreted in Sect. 5. Section 6 concludes.

## Theoretical framework

In this study we apply the dynamic-game framework (Basar and Olsder 1999; Basar and Zaccour 2018) in order to analyze coalition strategies between the countries in a monetary union facing different shocks. The economies under consideration are described by a dynamic system of nonlinear difference equations in state-space form:

1 $$\begin{aligned} x_t = f(x_{t-1},x_t,u_t^1,\dots ,u_t^N,z_t),\quad x_0={\bar{x}}_0. \end{aligned}$$

where $x_t$ is an ($n\times 1$) vector of state variables and $u_t^i$ is an ($m_i\times 1$) vector of individual control variables of player *i* ($i=1,\ldots , N$) having $m_i$ variables at their disposal. $z_t$ is a vector of non-controlled exogenous variables including exogenous shocks, $t=1,\ldots ,T$.

The problem is formulated in dynamic tracking game form where each player minimizes an objective function (loss function) $J^i$, which is the sum over time of quadratic deviations of state and control variables from given target values (denoted by $\sim$).

2 $$\begin{aligned} \min _{u_1^i,\ldots , u_T^i} J^i= \min _{u_1^i,\ldots , u_T^i}\sum _{t=1}^{T}L_t^i(x_t,u_t^1,\ldots , u_t^N), i=1,\ldots , N, \end{aligned}$$

with

3 $$\begin{aligned} L_t^i(x_t,u_t^1,\ldots , u_t^N)=[x_t-{\tilde{x}}_t^i]'\Omega _t^i[x_t-{\tilde{x}}_t^i] + [u_t-{\tilde{u}}_t^i]'\Psi _t^i[u_t-{\tilde{u}}_t^i]. \end{aligned}$$

The game is played for *T* periods and consists of individual optimization problems for *N* players. The penalty matrices $\Omega _t^i$ and $\Psi _t^i$ contain the weights of the deviations of states and controls from their desired levels in any period *t* and indicate the importance of each of the relevant variables for the players. Equation (3) allows each player to include the values of the other players’ control variables in their own objective function. However, in the present study only the players’ own control variables are considered (in addition to state variables).

Equations (1, 2, 3) define a nonlinear dynamic tracking game problem. Equilibrium solutions cannot be obtained analytically but are numerically approximated using the OPTGAME algorithm. This algorithm allows us to find approximations to cooperative (Pareto optimal) and non-cooperative Markov perfect (feedback) Nash equilibrium solutions of the game. A brief description of the algorithm is given below; for more details see Blueschke et al. (2013).
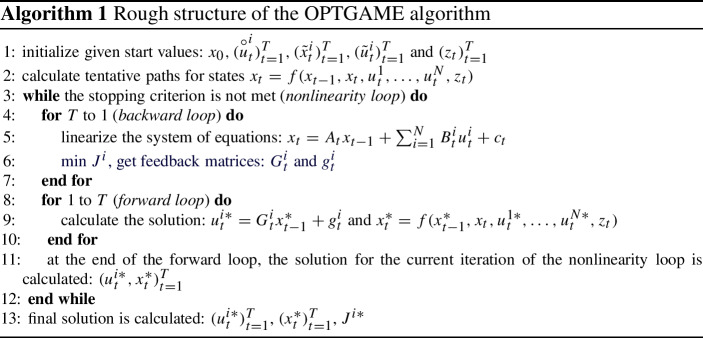


In step (1), all required data including the tentative paths of the control variables $(\overset{\circ }{u}_t^i)_{t=1}^T$ are included as inputs. In step (2), a tentative path for the state variables is calculated. The solutions of the nonlinear system of equations are obtained using trust region methods (alternatively, the Levenberg-Marquardt, Newton–Raphson or Gauss-Seidel method can be used). In the next step, the nonlinearity loop is initiated where an approximative solution of the nonlinear dynamic tracking game is obtained. To this end, the nonlinear system *f* is repeatedly linearized (step (5)) and solved using standard techniques along the tentative path determined in the previous step of the nonlinearity loop. Step (6) delivers the feedback matrices $G_t^i$ and $g_t^i$ by solving the dynamic tracking game for the linearized system. In steps (8—10) the solutions $u_t^{i*}$ and $x_t^{*}$ of the current iteration of the nonlinearity loop are calculated in a forward loop and, at the end of the nonlinearity loop, the final solutions are obtained. If the deviations of solutions of the current iteration from previous iteration is smaller than a pre-specified number, then the convergence criterion for the nonlinearity loop is fulfilled and the algorithm stops.

The optimization technique for minimizing the objective functions (step 6) depends on the type of the game or solution concept. For the current research two game strategies are considered: cooperative (Pareto optimal) and non-cooperative (Nash game for the feedback information pattern).

It must be stressed that the OPTGAME algorithm delivers only approximations to the true solutions of the nonlinear-quadratic game under consideration. Although we have tested OPTGAME for linear-quadratic dynamic games and confirmed that the (known) true solutions for this special case are obtained, the question of the quality of the approximations is an open one. This has some consequence for the problem of the uniqueness of the solutions, especially the Markov perfect Nash equilibrium solution. It is well known that this solution (the linear) is unique for linear-quadratic discrete-time dynamic games (see Basar and Olsder 1999, Corollary 6.1, pp. 280–281) but not necessarily so for linear differential and nonlinear dynamic games in general (see, for example, Tsutsui and Mino 1990). For them, general results are not available, although, as one referee pointed out, even in nonlinear games usually a generalization of the linear equilibrium strategy is picked up (see Rowat 2007). As our model is not highly nonlinear (with only one nonlinear equation), we trust the validity of the approximations and the sole consideration of the linear Markov-perfect or feedback Nash equilibrium strategy.

Another reservation concerns the neglect of stochastics in the calculations. A fully stochastic analysis for a dynamic game like ours would be enormously complicated as our experiences with the single-decision maker (optimal control) problem show (Blueschke et al. 2021). A mathematical analysis for the discrete-time case similar to the one by Basar and Haurie (1984) for the continuous-time case would be required, and the execution of stochastic simulations would be very cumbersome. An alternative would consist in considering re-optimizations at the beginning of each shock, assuming that the shock is unknown in advance but deterministic. This, as we have shown in another paper with a similar model (Blueschke et al. 2020), changes the noncooperative and cooperative strategies but with similar (and even more pronounced) trade-offs as in the present case. Finally, one may question the assumption of the finite time horizon and introduce a scrap value, which we found not to change the strategies significantly, apart from the last few periods. In view of these attempts, we consider the choice of a finite but longer than the effects of the shocks to be the most viable alternative. Our results therefore should not be interpreted as ex-ante advices to policy makers but as ex-post evaluations of past hypothetical policies.

## The model of the monetary union

The aim of this study is to analyze strategic economic interactions in a monetary union like the euro area in the presence of exogenous shocks such as the financial crisis, the Covid-19 pandemic, and the Ukraine war crisis. We have not modelled the exact situation in the euro area; rather we have created a simplified version of a monetary union with just three economies and a central bank. However, the monetary union is calibrated with the data of the euro area. To this end, we merge several euro area countries with more or less similar economic indicators of interest, which include public debt to GDP ratio, budget deficit, level of inflation and interest rate. We consider 19 euro area (EA) countries in our analysis and split them, in a first step, into two blocks: “core” and “periphery”. The core block consists of EMU countries with a more robust fiscal and inflation performance. The share of this block in the EA’s real GDP was 60% in 2007 (pre-financial crisis situation). The periphery block consists of countries with higher public debt and/or deficits as well as higher interest and inflation rates on average. The share of this block in the EA’s real GDP was 40% in 2007.

In a second step the periphery block is split into two sub-blocks. We assume that, despite their similar initial economic situation (first of all higher public debt compared to the core block), the periphery block is not homogenous regarding the importance of fiscal stability indicators. To this end we create a sub-block with more thrifty economies, i.e. fiscal variables are given higher importance/values in the weighting matrices $\Omega$ and $\Psi$ (see Eq. (2, 3), and a sub-block with less thrifty economies. As this information is not directly observable for real EA economies, we simply split the periphery block into two equal parts each having a 20% share in the real GDP of the monetary union. Altogether we consider four policy makers:

**Table:** 

Acronym	Player	Calibration for Euro area
C1	Government of country 1	Core block
C2	Government of country 2	Thrifty periphery block
C3	Government of country 3	Less thrifty periphery block
CB	Common central bank	ECB

The governments’ decisions represent fiscal policy and the common central bank of the monetary union is responsible for controlling monetary policy. The common central bank decides on the prime rate $R_{Et}$, a nominal rate of interest under its direct control. The national governments decide on real fiscal surplus (or, if negative, its fiscal deficit), $g_{it}$ $(i=1, 2, 3)$, measured in relation to real GDP. The players use their control variables as instruments in order to track the desired paths of the state variables, which evolve according to the dynamic system. Table  shows the list of state variables considered in our study.

**Table 1:** Variables of the three-country (*i*=1,...,3) monetary union

Control variables	
$g_{it}$	Real fiscal surplus of country *i*
$R_{Et}$	Prime rate
Endogenous variables	
$y_{it}$	Short-term deviations from the LR equilibrium output level
$r_{it}$	Real interest rate in country *i*
$I_{it}$	Nominal interest rate in country *i*
$\pi _{it}$	Inflation rate in country *i*
$\pi ^{e}_{it}$	Expected inflation rate in country *i*
$y_{Et}$	Weighted output in the monetary union
$\pi _{Et}$	Weighted inflation rate in the monetary union
$D_{it}$	Real government debt in country *i*
$BI_{it}$	Average interest rate for government bonds in country *i*

The dynamic model, which describes the evolution of the state variables over time, is formulated in terms of deviations from a long-run growth path and consists of the following equations:

4 $$\begin{aligned} y_{it}= & {} \delta _i\left( \dfrac{\pi _{jt}+\pi _{kt}}{2}-\pi _{it}\right) -\gamma _i(r_{it}-\theta )\nonumber \\{} & {} +\rho _{ij}y_{jt}+\rho _{ik}y_{kt}-\beta _i\pi _{it}+\kappa _i y_{i,t-1}-\eta _i g_{it}+ zd_{it}, \end{aligned}$$

5 $$\begin{aligned} r_{it}= & {} I_{it}-\pi _{it}^e, \end{aligned}$$

6 $$\begin{aligned} I_{it}= & {} R_{Et}-\lambda _i g_{it} + \chi _i D_{it}, \end{aligned}$$

7 $$\begin{aligned} \pi _{it}= & {} \pi _{it}^e +\xi _i y_{it} + zs_{it}, \end{aligned}$$

8 $$\begin{aligned} \pi _{it}^e= & {} \varepsilon _i\pi _{i,t-1}+(1-\varepsilon _i)\pi _{i,t-1}^e, \varepsilon \in [0,1], \end{aligned}$$

9 $$\begin{aligned} y_{Et}= & {} \sum _{i=1}^3\omega _i y_{it}, \sum _{i=1}^3\omega _i=1, \end{aligned}$$

10 $$\begin{aligned} \pi _{Et}= & {} \sum _{i=1}^3\omega _i \pi _{it}, \sum _{i=1}^3\omega _i=1, \end{aligned}$$

11 $$\begin{aligned} D_{it}= & {} (1+BI_{i,t-1}-\pi _{i,t-1}^e)D_{i,t-1}-g_{it}, \end{aligned}$$

12 $$\begin{aligned} BI_{it}= & {} \frac{1}{6}\sum _{\tau =t-5}^t I_{it}. \end{aligned}$$

The goods markets are modelled by the short-run income-expenditure equilibrium relation (Eq. 4) for real output $y_{it}$. $\theta \in [0,1]$ is the natural real rate of output growth and is assumed to be equal to the natural real rate of interest. Excess demand for goods and services depends on the domestic inflation rate relative to that in the other two countries, on the real rate of interest relative to the natural rate, on aggregate excess demand in the other two countries (through exports into them), on the domestic inflation rate (a Pigou-Haberler effect) and on the domestic budget surplus/deficit (through a Keynesian multiplier). Exogenous demand-side shocks can affect the domestic output via $zd_{it}$.

The current real rate of interest $r_{it}$ is given by the Fisher equation (Eq. 5). The nominal rate of interest $I_{it}$ (Eq. 6) is driven by the prime rate, adjusted by country-specific risk premiums $-\lambda _i$ and $\chi _i$. Hence, the nominal rates of interest can be different in the monetary union despite a common monetary policy. The inflation rate $\pi _{it}$ is determined by an expectations-augmented Phillips curve (Eq. 7), in which the expected rate of inflation is based on adaptive expectations. Exogenous supply-side shocks (such as energy price shocks) can enter the inflation equation through $zs_{it}$.

The real government debt $D_{it}$ measured in relation to GDP evolves according to the government budget equation (Eq. 11) and depends on the previous stock of public debt, the current budget surplus/deficit and interest payments that depend on the interest rate on bonds $BI_{it}$. An average government bond maturity of six years (Eq. 12) is assumed following Krause and Moyen (2016). Equation (11) is the only nonlinear in our model. We do not linearize it because the multiplicative relation between the endogenous variables government debt and interest rate is essential for the development of the trade-off between output, price stability and public debt and the possibility of exploding public debt relevant for the euro area during the period of interest.

The average values of output and union-wide inflation in the monetary union are given by Eqs. (9, 10). The parameter $\omega _i$ in these equations expresses the weight of country (block) *i* in the economy of the whole monetary union as defined by its output level.

The parameters of the model are calibrated for the long-run growth path and are given in Table .

**Table 2:** Parameter values for an asymmetric monetary union, $i=1,2,3$

						$$\rho _{23},\rho _{32}, \beta _i$$			
*T*	$\theta$	$\omega _1$	$\omega _2,\omega _3$	$\delta _i,\eta _i,\varepsilon _i$	$\rho _{21},\rho _{31}$	$\gamma _i,\kappa _i,\lambda _i$	$\rho _{12},\rho _{13}$	$\xi _i$	$\chi _i$
30	3	0.6	0.2	0.5	0.375	0.25	0.125	0.1	0.0125

As can be seen from Table 2 we consider a game with a finite time horizon, namely 30 periods, interpreted here as years. The long-run growth rate $\theta$ is assumed to be 3%. The economic weights of the players $\omega _i$ correspond to the EA’s real GDP in 2007 for the considered blocks of countries with the core block having 60% weight and the two periphery blocks having 20% each.

The dynamic system as given by Eqs. (4–12) describes the evolution of the state variables over time. The four players use their control variables to lead the objective state variables in the desired direction (minimizing quadratic deviations). However, the players are different with respect to the set of state variables in their objective function as well as to the importance of the individual objective variables. As can be seen in Eq. (13) governments ($i=1,2,3$) look upon their national variables: inflation, output, public debt and budget balance. In contrast, the common central bank (*E*, Eq. 14) looks upon union-wide inflation, average output in the monetary union and the prime rate.

13 $$\begin{aligned} J_i= & {} \frac{1}{2}\sum _{t=1}^{T}\left( \frac{1}{1+\frac{\theta }{100}}\right) ^t\{\alpha _{\pi i}(\pi _{it}-{\tilde{\pi }}_{it})^2+\alpha _{yi}(y_{it}-{\tilde{y}}_{it})^2\nonumber \\{} & {} +\alpha _{Di}(D_{it}-{\tilde{D}}_{it})^2+\alpha _{gi}g_{it}^2\} \end{aligned}$$

14 $$\begin{aligned} J_E= & {} \frac{1}{2}\sum _{t=1}^{T}\left( \frac{1}{1+\frac{\theta }{100}}\right) ^t\{\alpha _{\pi E}(\pi _{Et}-{\tilde{\pi }}_{Et})^2\nonumber \\{} & {} +\alpha _{yE}(y_{Et}-{\tilde{y}}_{Et})^2+\alpha _{E}(R_{Et}-{\tilde{R}}_{Et})^2\} \end{aligned}$$

The governments have the same set of state variables but there is an asymmetry in the importance of the variables to the policy maker concerned as given by parameter $\alpha$ (compare Table ). The main difference between the governments in this regard is the importance of the public debt target ($\alpha _D$) in the objective function. Country 1 and country 2 (oriented towards fiscal stability) attach a ten times higher weight to it than country 3 (the less thrifty periphery block). In addition, there is a clear difference between governments and the common central bank. The governments put higher emphasis on (national) output while the central bank gives a higher weight to (union-wide) inflation.

**Table 3:** Weights of the variables in the objective functions

$\alpha _{yi}, \alpha _{gi}$	$\alpha _{\pi E}$	$\alpha _{yE},\alpha _{\pi i}$	$\alpha _{D1},\alpha _{D2}$	$\alpha _{D3}$	$\alpha _{RE}$
1	5	0.5	1	0.1	3

Finally, the desired paths of the objective variables have to be defined. These target values are summarized in Table . A balanced growth path (the natural level of real GDP) is targeted by all players, i.e. the short-run output gap should be zero (${\tilde{y}}_i=0$). The target value for the inflation rate is set to 2%, which corresponds to the official objective of the ECB. Regarding the public debt target, the governments aim to fulfill the Maastricht criteria of 60% of GDP, taking different initial values into account. As the periphery blocks start from a higher level, they steer towards a linear decrease in public debt from 80% to 60% over the entire planning horizon. Finally, the governments prefer a balanced budget (${\tilde{g}}_i=0$) and the central bank aims at a prime rate of 3%. The target values and the weights given to the policy instruments of each player partly reflect the desire to avoid too excessive fluctuations in these variables, which are not possible in the real world due to the path dependence of policies.

**Table 4:** Target values for the asymmetric monetary union

${\tilde{D}}_{1t}$	${\tilde{D}}_{2t},{\tilde{D}}_{3t}$	${\tilde{\pi }}_{it}$	${\tilde{\pi }}_{Et}$	${\tilde{y}}_{it}$	${\tilde{y}}_{Et}$	${\tilde{g}}_{it}$	${\tilde{R}}_{Et}$
60	80$\searrow$60	2	2	0	0	0	3

We obtain a non-cooperative Nash equilibrium and a cooperative Pareto solution for the considered game. For the cooperative Pareto scenario, the joint objective function is given by the equally weighted sum of the four objective functions:

15 $$\begin{aligned} J=\mu _1J_1 + \mu _2J_2 + \mu _3J_3 +\mu _EJ_E, (\mu _1=\mu _2=\mu _3=\mu _E=0.25). \end{aligned}$$

In order to check for the sensitivity of the Pareto solution with respect to the weights of the players, we also determined a Pareto solution with equal weights (0.5) for monetary policy (the central bank) and fiscal policy (the governments in a body), which gave results similar to those reported here (with more active fiscal and less active monetary policy). In any case, the Pareto solution requires full commitment of all the players, which would have to be guaranteed by appropriate institutional devices. Using the dynamic system (4–12) and the objective functions (13–14) we are able to analyze growth, fiscal stability and price stability trade-offs in a monetary union in the presence of exogenous shocks. The considered model is called MUMOD2. It is calibrated for the euro area and is solved using the OPTGAME3 algorithm (see Blueschke et al. 2013).

## Exogenous shocks

The MUMOD2 model allows for a game-theoretic analysis of economic interactions in a monetary union like the euro area in the presence of exogenous shocks. In this study we emulate certain aspects of the financial and economic crisis 2008–2010, the ensuing European sovereign debt crisis, the Covid-19 crisis, and the Ukraine war crisis. We follow the argumentation of Kahle and Stulz (2013) and model the financial and economic crisis as a demand shock. To be more precise we add an exogenous shock on the demand side with a drop in GDP by 1% in 2008, by 6% in 2009 and by 1% in 2010. We model this shock in a symmetric way to all economies in the monetary union. In contrast, the ensued sovereign debt shock impacts the periphery block only. The numerical values of these shocks are summarized in Table .

**Table 5:** Modelling the financial crisis and the sovereign debt crisis

*t*	1	2	3	4	5	6	7	8	9	$\cdots$
Year	’08	’09	’10	’11	’12	’13	’14	’15	’16	$\cdots$
$zd_{1t}$	$-1$	$-6$	$-1$	0	0	0	0	0	0	0
$zd_{2t}$	$-1$	$-6$	$-1$	$-3$	$-4$	$-3$	$-1$	0	0	0
$zd_{3t}$	$-1$	$-6$	$-1$	$-3$	$-4$	$-3$	$-1$	0	0	0

In contrast to the financial crisis and the sovereign debt crisis shocks which hit the euro area mainly via the demand side, the Covid-19 shock is not just a negative demand-side shock. In addition to the restrictions on private consumption and investment, the pandemic situation interrupted existing supply chains, leading to increasing production costs, and can be modelled as a supply-side shock as well; in addition, higher prices of oil and other resources have similar effects on the euro area. For a discussion of the different channels of the Covid-19 shock see del Rio-Chanona et al. (2020). Following this line of argumentation, we interpret the Covid-19 shock as a combination of demand- and supply-side shocks in our study. Although the pandemic is still (end of 2022) ongoing, we model the strongest negative impact of the shock on the demand side in 2020. The last available forecast for the volume of world trade is from 12 April 2022 from the WTO, estimating a 5% drop in 2020 with an increase of 9.8% in 2021 and increases of around 3% in the following years. In contrast the supply-side effects start to impact the economies under consideration in 2021 and, amplified by the Ukraine war, starting in 2022, slowly decreasing over the next three years and completely disappearing in 2025. The Ukraine war crisis is still ongoing at the time of writing this paper. We interpret it as a supply shock reinforcing and extending the supply-side elements of the Covid-19 shock. The numerical values of the Covid-19 and the Ukraine war shocks are summarized in Table .

**Table 6:** Modelling the Covid-19 crisis and the Ukraine war crisis

*t*	12	13	14	15	16	17	18	19	20	21	$\cdots$
Year	’19	’20	’21	’22	’23	’24	’25	’26	’27	’28	$\cdots$
$zd_{1t}$	0	$-5$	0	0	0	0	0	0	0	0	0
$zd_{2t}$	0	$-5$	0	0	0	0	0	0	0	0	0
$zd_{3t}$	0	$-5$	0	0	0	0	0	0	0	0	0
$zs_{1t}$	0	0	1	9	3	2	1	0	0	0	0
$zs_{2t}$	0	0	1	9	3	2	1	0	0	0	0
$zs_{3t}$	0	0	1	9	3	2	1	0	0	0	0

Summarizing, we include different shocks on the monetary union in our study:

1. A negative demand-side shock in the goods markets due to the financial and sovereign debt crises 2008–2016 (represented by the variable $zd_{it}$ in Table 5)
2. A negative demand-side shock due to the Covid crisis 2020 ($zd_{it}$ in Table 6 )
3. A negative supply-side shock due to the Covid and Ukraine war crises 2021–2025 ($zs_{it}$ in Table 6).

## Results

The aim of this research is to analyze the effects of different coalition strategies in a monetary union in the presence of negative exogenous shocks. We consider three fiscal players with asymmetric initial situations and objective functions and a common central bank responsible for monetary policy. Five scenarios are established with different coalitions (as summarized in Table ). By a coalition, we mean a strictly binding agreement between two or more players to always act jointly, i.e. the participants in the coalition give up their own objective function and play as one player (following a cooperative strategy inside the coalition) with a weighted joint objective function.

*sc1_NF4* denotes an “everyone for themselves” scenario. This means a non-cooperative Nash game with all four players being independent, i.e. each player only cares about their own objective function. *sc2_2+3* denotes a non-cooperative Nash game with three players, where country 2 and 3 build a coalition. It means that the central bank (CB) plays against country 1 (core block) and against a coalition of countries 2 and 3 (periphery block). *sc3_1+2* denotes a Nash game with three players, where the countries oriented towards fiscal stability form a coalition and play together against the central bank and country 3. *sc4_FU* denotes a Nash game with two players, where a coalition of all fiscal players is formed. This strategy corresponds to the creation of a fiscal union with an independent central bank. *sc5_P* stands for total integration of fiscal and monetary policy and presents a fully cooperative Pareto solution. Moreover, we consider the non-controlled forward simulation using the starting values of the control variables (*sim*).

**Table 7:** Coalition strategies when facing negative exogenous shocks

	Scenario	Game strategy
	Everyone for themselves	Nash FB with 4 players
	Core vs periphery	Nash FB with 3 players
	Coalition of periphery countries	CB / C1 / (C2+C3)
sc3_1+2	Thrifty vs thriftless	Nash FB with 3 players
	Coalition of countries 1 & 2	CB / (C1+C2) / C3
	Fiscal union	Nash FB with 2 players
	Coalition of 3 countries	CB / (C1+C2+C3)
	Fiscal and monetary union	Pareto solution
	Non-controlled simulation	Simulation

The task is to compare the performances of the players for different coalition scenarios. To this end we present graphs for the control and three state variables, output, inflation rate and public debt. In addition, the performance of individual players and coalitions is given by the resulting objective function values (loss functions to be minimized) in Table .

Figure  shows the outcomes for the control variables (prime rate and fiscal surplus) of the players. Figures , and   present the results of the state variables (output and public debt).

**Fig. 1:**
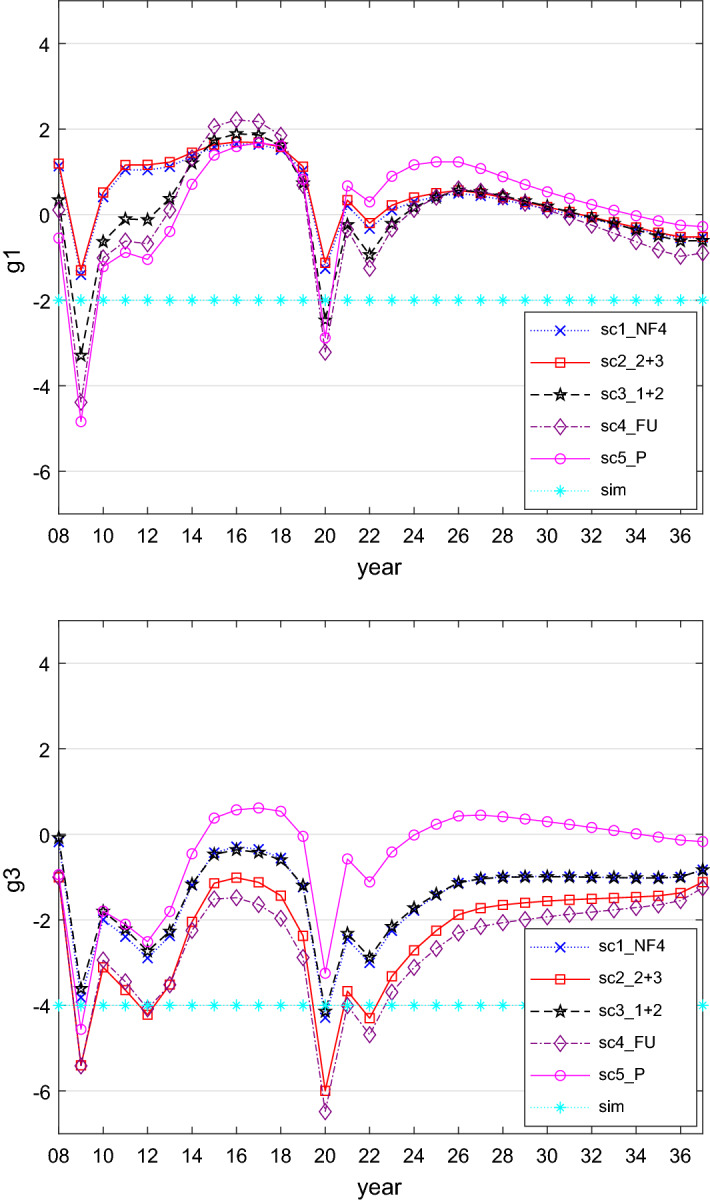
Control variables (prime rate ($R_{Et}$) and fiscal surplus ($g_{1t}, g_{2t}, g_{3t}$))

**Fig. 2:**
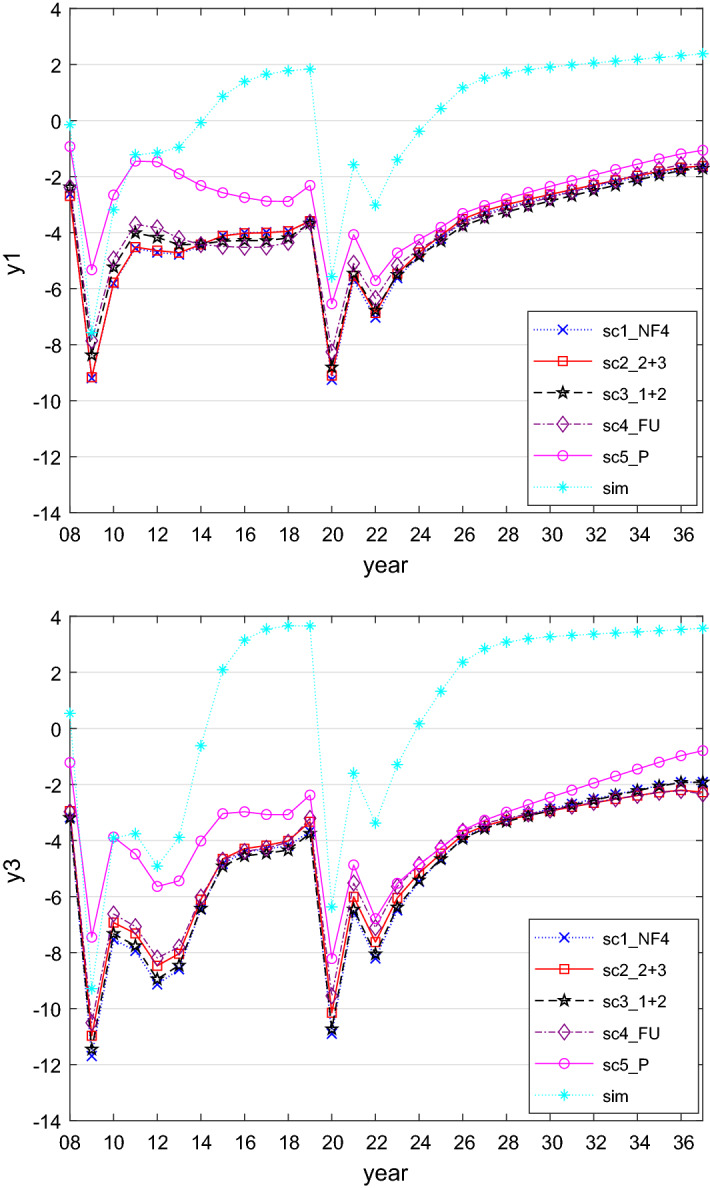
Country *i*’s (and union-wide average) output $$y_{1t}, y_{2t}, y_{3t}, y_{Et} $$

**Fig. 3:**
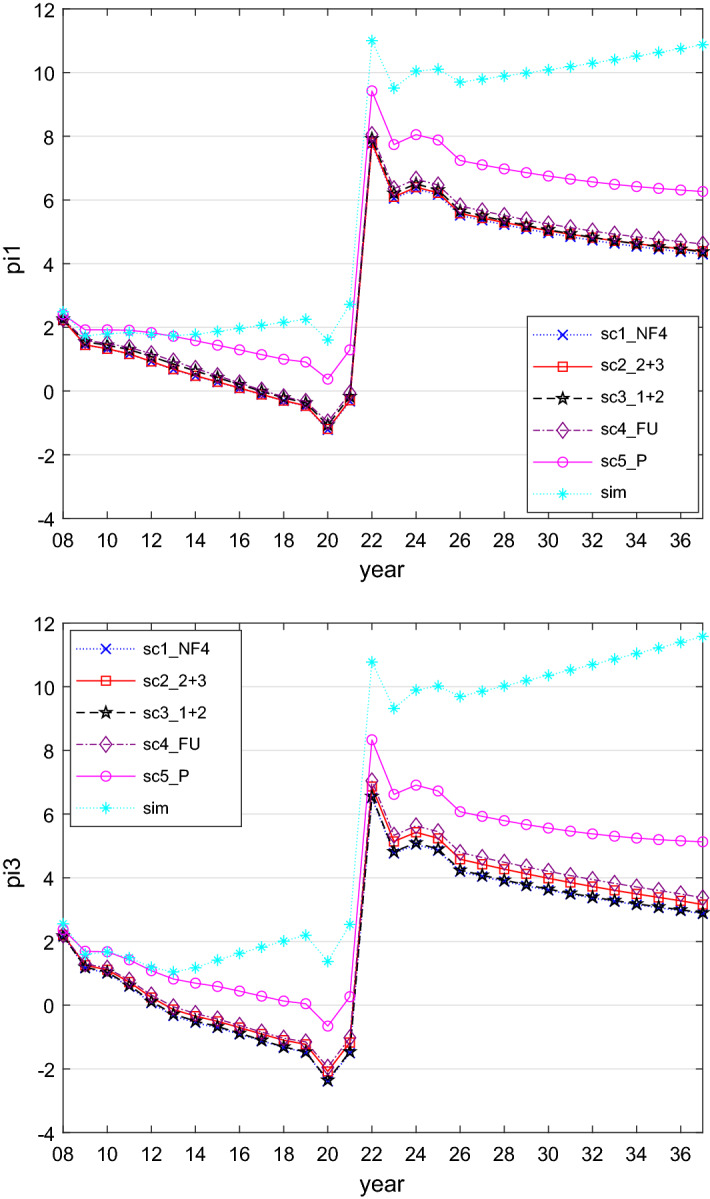
Country *i*’s (and union-wide average) inflation $\pi _{1t}, \pi _{2t}, \pi _{3t}, \pi _{Et}$

**Fig. 4:**
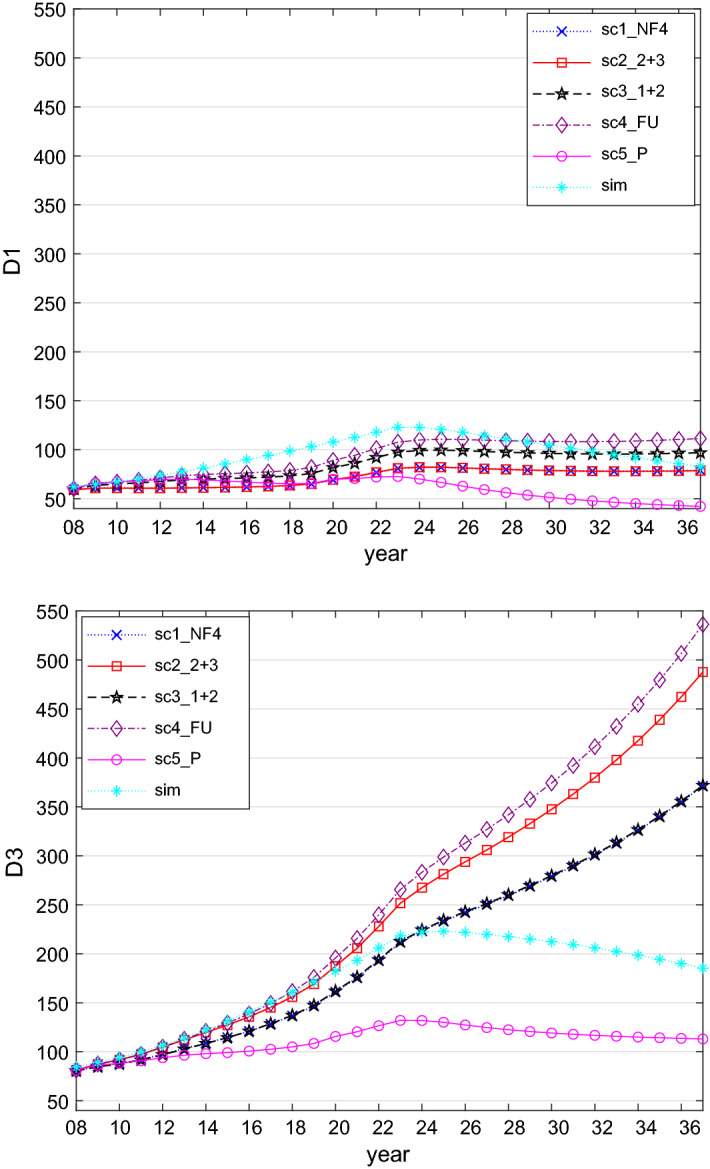
Country *i*’s debt level $D_{1t}, D_{2t}, D_{3t}$

**Table 8:** Objective function values

sc	CB	C1	C2	C3
sc1_NF4	479.89	290.44	624.26	489.81
sc2_2+3	489.52	286.50	628.08	523.85
sc3_1+2	477.30	329.33	620.24	478.14
sc4_FU	500.09	374.85	618.30	527.37
sc5_P	624.36	193.11	277.63	249.29
sim	1522.25	397.80	1591.95	481.42
sc	C1+C2	C2+C3	C1+C2+C3	CB+C1+C2+C3
sc1_NF4	914.70	1114.07	1404.51	**1884**.**39**
sc2_2+3	914.58	1151.93	1438.43	**1927**.**95**
sc3_1+2	949.57	1098.38	1427.72	**1905**.**02**
sc4_FU	993.15	1145.66	1520.51	**2020**.**61**
sc5_P	470.75	526.92	720.03	**1344**.**40**
sim	1989.75	2073.37	2471.17	**3993**.**41**

The results allow the following insights from this analysis:

1. The policy instruments are used in a countercyclical way during the demand shocks, which is in line with the underlying Keynesian dynamic model. The two demand-side shocks have similar effects, while the deeper Covid-19 shock requires a more active monetary and fiscal intervention by all players. However, it is interesting to note that monetary policy and mostly also fiscal policy return faster to their “business as usual” course, which is in contrast to the extremely expansionary policy the European Central Bank actually pursued over an extended period after the financial crisis. Fiscal policies, at least of the thrifty countries, not only return to the log-run steady state (0 in our model) but overshoot by producing primary surpluses after the end of the demand shocks, taking care of the sustainability of their public debt. Moreover, neither monetary nor fiscal policy react to the supply-side shock in the last phase of the Covid-19 shock. Thus, even this Keynesian model does not support longer expansionary phases of countercyclical policies after temporary demand shocks or any expansionary policy against a supply shock. This is a feature that agrees with results from new-classical or monetarist models.
2. In accordance with the dynamic games literature, the cooperative Pareto solution results in the best performance in terms of the value of the overall objective function. Table 8 and the graphs clearly show that the main burden of cooperation falls on the common central bank, which helps the economies to mitigate the negative effects of the crises by running an expansionary monetary policy. This situation corresponds to the actual policy of the ECB in the last few years, apart from the more discretionary reaction of monetary policy our model calls for. An accommodating monetary policy therefore provides an important advantage to fiscal policy makers, as we have already shown in Blueschke and Neck (2018). In the cooperative Pareto solution, all countries perform much better in terms of both output and public debt than in any of the other scenarios. This is true despite the fact that in such a situation they run the highest budget deficits nearly all of the time, compared to other coalition strategies. Herein lies one of the main advantages of cooperation as all players have binding agreements and know that no one would beggar their neighbour. The accommodation of this expansionary fiscal policy stance by the joint monetary policy makes them more effective and allows for more expansionary actions than otherwise.
3. Comparing both periphery countries, one can see that country 3 runs much higher fiscal deficits, which is explained by its assumed lower preference for fiscal stability. However, this leads to unsustainable public debt levels in all scenarios other than the cooperative Pareto solution. This would result in the bankruptcy of this country by the end of the planning horizon. We do not include the possibility of bankruptcy or a hair-cut in this study; that issue was analyzed in a previous study indicating the possibility of negative impacts of hair-cuts for all players (for more details see Neck and Blueschke 2014). On the other hand, country 2, which accords higher importance to fiscal stability, is able to stabilize its public debt at a sustainable level even after four consecutive crises in the last 14 years. This indicates the necessity for a careful design of fiscal policy especially in periphery countries, with an emphasis on the goal of government debt sustainability in order to avoid a situation leading to another European sovereign debt crisis.
4. The analysis of strategies in different coalition scenarios allows us to obtain interesting insights into the underlying dynamics. The pure fiscal union scenario ($sc4\_FU$ with all fiscal players being part of the same coalition) gives the worst solution in terms of the total objective function value. However, in this scenario the main burden of mitigating the exogenous shocks falls on the fiscal players running very high budget deficits during the shocks. In contrast, the central bank does not really support the fiscal players in this scenario and instead chooses high prime rates in order to fulfil its primary mandate to secure price stability. This shows that a pure fiscal union, without accommodating monetary policy, can deliver results that are worse than even the scenario without any coordination at all. This result is new and points toward the need for coordination not only between the governments but also between them and the central bank. Creating a finance minister or some other form of a fiscal union without arrangements for coordinating with the monetary authorities may lead to a situation where fiscal and monetary policies counteract each other and may be highly inefficient.

Comparing both ’small’ coalition strategy scenarios ($sc2\_2+3$ and $sc3\_1+2$), one can see that the country which allies itself with the country with the higher government debt has to apply a more active fiscal policy than otherwise. In the case of $sc2\_2+3$ (the coalition of periphery countries) this is true for country 2 and in the case of $sc3\_1+2$ (the coalition of core and thrifty periphery countries) for country 1. Being part of a fiscal coalition allows country 3 to run higher deficits and to concentrate more on the growth side of the growth-public debt trade-off. On the contrary, if left alone, this country has to pay more attention to the public debt target. This behaviour results in significantly lower public debt levels in country 3 in the scenarios where it does not cooperate ($sc1\_NF4$ and $sc3\_1+2$). Again, this result raises doubts about the idea of a fiscal union as a possible solution for unsustainable high public debts in some euro area countries.

## Conclusions and outlook

In this paper, we analyzed the dynamics in a monetary union consisting of three asymmetric fiscal players and a common central bank in the presence of exogenous shocks. The monetary union was calibrated for the euro area, starting at the pre-financial crisis level, and the shocks were modelled to imitate the negative effects of the financial crisis, the European sovereign debt crisis, the Covid-19 crisis and the Ukraine war crisis. We calculated cooperative and non-cooperative Nash equilibrium solutions for different coalition strategy scenarios. The fully cooperative (Pareto) solution gives the best results in terms of the objective function values and requires a very active monetary policy. The fiscal union coalition scenario without cooperation with the central bank gives the worst outcome (in terms of the overall objective function) of all scenarios examined because the central bank thwarts the governments endeavours. This scenario requires active fiscal policy by all fiscal players but this results in extremely high public debt levels which, especially for country 3, lead eventually to bankruptcy. This is true as well for the periphery block coalition scenario.

Altogether, the results confirm the general insight that the cooperation of policy makers may improve the outcome but this cooperation has to be comprehensive in taking all relevant players on board. Partial coalitions may result in losses to some or even all policy makers involved. The centralization of fiscal policies for stabilization purposes (a fiscal union) can therefore be recommended only if it is also coordinated with monetary policy.

These results can have some interpretations in terms of current policy discussions, for instance about the possibility of endowing the euro group with a “European finance minister” and a separate budget. In order to relate them more closely to these debates, the analysis would have to be augmented by a more elaborate macroeconomic model and a more sophisticated calibration of the model. A more detailed calibration of the euro area, especially with a higher number of countries, would also be required and is the aim for future research.
